# Picture quiz

**Published:** 2019-02-10

**Figure:**
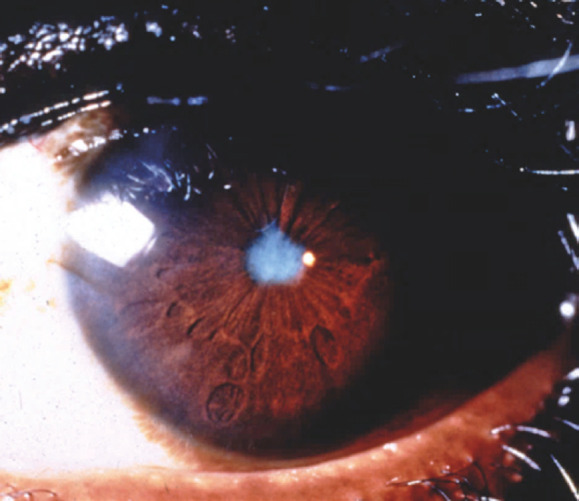


A 40-year-old man presents with poor vision. The left eye has phthisis bulbi with no light perception. The right eye has perception of light with accurate projection of light.

See picture above.

**Question 1**

**What do you see on examination?**

**Question 2**

**How could this have been prevented?**

**Question 3**

**What are the principles involved in management?**

## ANSWERS

1. The cornea appears clear. The pupil is small and irregular. The pupil is white, probably due to a fibrotic membrane; however, it may also be due to lens opacity. From this picture it is not possible to assess the depth of the anterior chamber, but if the pupil is occluded and aqueous cannot pass into the anterior chamber then the peripheral iris may be pushed forward.
2. The original problem is almost certainly iritis which has caused adhesions between iris and lens, also known as posterior synechiae. The adhesions could have been prevented if the pupil had been dilated at the original episode of iritis and kept dilated until the iritis resolved.
3. This is a complicated case to manage. It is the patient's only functioning eye. The patient can see light with accurate projection, which indicates that the optic nerve and retina are functioning.
  The principles involved in management are:
  - Check the intraocular pressure and, if raised, lower it either medically or with a laser or peripheral iridotomy
  - Break the adhesions causing the small irregular pupil; mydriatics can be tried first. If this does not work, then surgery will be indicated.
  - When the pupil adhesions have been broken and any membrane removed the lens can be assessed. If clear, no further surgical treatment is indicated; if there is a cataract, then this will have to be removed. Surgical management of the small irregular pupil and removal of a cataract (if present) is difficult; it should only be undertaken by an ophthalmologist with experience in managing patients with complicated cataract.

